# Methodological considerations for and validation of the ultrasonographic determination of human skeletal muscle hypertrophy and atrophy

**DOI:** 10.14814/phy2.14683

**Published:** 2021-01-06

**Authors:** Tanner Stokes, Thomas R. Tripp, Kevin Murphy, Robert W. Morton, Sara Y. Oikawa, Hon Lam Choi, Jessica McGrath, Chris McGlory, Maureen J. MacDonald, Stuart M. Phillips

**Affiliations:** ^1^ Department of Kinesiology McMaster University Hamilton Ontario Canada; ^2^ Faculty of Kinesiology University of Calgary Alberta Canada; ^3^ School of Kinesiology and Health Sciences Queen’s University Kingston Ontario Canada

**Keywords:** exercise, human, imaging, immobilization

## Abstract

Magnetic resonance imaging (MRI) is the current gold standard for measuring changes in muscle size (cross‐sectional area [CSA] and volume) but can be cost‐prohibitive and resource‐intensive. We evaluated the validity of B‐mode ultrasonography (US) as a low‐cost alternative to MRI for measuring muscle hypertrophy and atrophy in response to resistance training and immobilization, respectively. Fourteen young men performed 10wk of unilateral resistance training (RT) to induce muscle hypertrophy. In the final two weeks of the 10wk, the subjects’ contralateral leg was immobilized (IMB). The cross‐sectional area of the *vastus lateralis* (VLCSA) was measured at the mid‐thigh before and after each intervention using MRI (VLCSA_MRI_) and US (VLCSA_US_). The relationship and agreement between methods were assessed. Reliability of US measurements ranged from good to excellent in all comparisons (ICC >0.67). VLCSA significantly increased after 10 weeks of RT (VLCSA_US_: 7.9 ± 3.8%; VLCSA_MRI_: 7.8 ± 4.5%) and decreased after 2 weeks of IMB (VLCSA_US_: −8.2%±5.8%; VLCSA_MRI_: −8.7 ± 6.1%). Significant correlations were identified between MRI and US at each time point measured (all r > 0.85) and, importantly, between MRI‐ and US‐derived changes in VLCSA. Bland‐Altman analysis revealed minimal bias in US measurements relative to the MRI (−0.5 ± 3.0%) and all measurements were within the upper and lower limits of agreement. Our data suggest that B‐mode ultrasonography can be a suitable alternative to MRI for measuring changes in muscle size in response to increased and decreased muscle loading in young men.

## Graphical abstract

We demonstrate that ultrasound‐derived measurements of vastus lateralis hypertrophy and atrophy induced by resistance training and immobilization are significantly correlated with MRI‐derived measurements. The reliability of our ultrasound technique was also in the range of good‐excellent. Together, these data suggest that B‐mode ultrasonography is a suitable alternative for the assessment of muscle hypertrophy and atrophy under conditions of dynamic loading in young men.

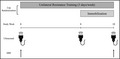

## 1 INTRODUCTION

Skeletal muscle generates the forces necessary to perform activities of daily living and is a reservoir of amino acids that can be mobilized to supply gluconeogenic substrates during catabolic states (Wolfe, 2006). Muscle atrophy occurs with aging (Janssen et al., 2000, 2002), muscle disuse (Glover et al., 2008; Holloway et al., 2019; Yasuda et al., 2005), and/or disease (Powers et al., 2016), and is associated with functional impairment and metabolic dysregulation. Resistance training (RT) is potently anabolic and results in hypertrophy (Stokes et al., 2020). Additionally, RT is an effective non‐pharmacological strategy to prevent or attenuate muscle atrophy during catabolic conditions (Devries et al., 2015; Fernandez‐Gonzalo et al., 2020; Moore et al., 2018; Oates et al., 2010), in part due to its potent hypertrophic effect on skeletal muscle.

A reproducible observation with both increased and decreased loading of skeletal muscle is the substantial interindividual heterogeneity of the hypertrophic response with loading (Davidsen et al., 2011; Hubal et al., 2005; Stokes et al., 2020) and atrophy with unloading (Chen et al., 2017; Glover et al., 2008; Stokes et al., 2020; Yasuda et al., 2005). Interestingly, the ability to accurately quantify changes in muscle size in response to hypertrophy‐inducing loading has recently been identified as being more complex than once thought, with substantial variability between methods (Haun et al., 2019). We propose a similar critique would be true when describing the response to unloading‐induced atrophy. Microscopic assessments of myofiber cross‐sectional area (CSA) are the only available method for the assessment of muscle fiber size; however, few laboratories have the capacity to obtain biopsy specimens and the invasive nature of the procedure may preclude measurements in certain populations (i.e., the frail elderly; (Wilson et al., 2018). These difficulties inevitably complicate the comparison of research findings between laboratories. Thus, cheaper and more readily accessible, non‐invasive methods to assess muscle hypertrophy and atrophy are warranted.

Magnetic resonance imaging (MRI) is a non‐invasive and non‐radioactive means to determine muscle CSA (and volume) that offers unparalleled tissue differentiation capabilities and has a high correlation with the cadaver‐measured determination of muscle (Mitsiopoulos et al., 1998). For these reasons, MRI is considered the gold standard for assessing changes in muscle size. However, obtaining MRI images requires highly specialized personnel and is otherwise limited by operation cost, availability, and time‐consuming post‐acquisition image processing. A potential low‐cost and more readily available alternative to MRI is B‐mode ultrasonography (US)(Parker et al., 2008). US has been shown to accurately quantify muscle CSA in both young (Franchi, Longo, et al., 2018; Lixandrao et al., 2014) and older adults (Nijholt et al., 2017; Reeves et al., 2004) when measured at a single‐time point. Moreover, changes in muscle thickness obtained by US correlated with MRI‐derived changes in anatomical CSA, but not muscle volume, after 12 weeks of isokinetic RT (Franchi, Longo, et al., 2018).

No study, to date, has assessed the efficacy of US, relative to MRI, to accurately capture changes in *vastus lateralis* CSA (VLCSA)—a muscle routinely sampled by needle biopsy—before and after muscle hypertrophy and atrophy‐inducing stimuli. It is plausible that a two‐dimensional assessment of muscle size (i.e., VLCSA) will produce stronger correlations between MRI‐derived measurements of VLCSA and, perhaps, muscle volume compared to unidimensional thickness measurements. Thus, the purpose of this study was to evaluate the relationship and methodological agreement between US and MRI measured *changes* in VLCSA after 10 weeks of unilateral RT and 2 weeks of brace‐mediated immobilization (IMB). We hypothesized that US would show good concordance with the criterion measure for muscle size, MRI, showing similar changes in VLCSA in response to RT (hypertrophy) and immobilization (atrophy).

## 2 METHODS

### 2.1 Participants for methodological development

Prior to conducting the main study, we were interested in objectively quantifying the effect of applied force on an ultrasound probe to derive muscle thickness measurements. We studied 34 male participants from a previously published investigation (Age, 23 ± 3 yrs; Body Mass, 86.6 ± 13.5 kg; BMI, 26.4 ± 3.3 kg/m^2^; (Morton et al., 2016)). Muscle thickness measurements were taken, as described below (see *Ultrasonography)*, from the *vastus lateralis* prior to and upon the completion of 12 weeks of whole‐body resistance training at four different applied probe forces (0.2, 0.5, 0.7, and 1.0 N).

### 2.2 Participants for validation trial

For a detailed description of the participant recruitment strategy and screening procedures for the main trial, please refer to a previous report from the same participants (Stokes et al., 2020). Briefly, 14 recreationally active men (18–30 y) who were free of chronic disease and/or not currently taking medications known to influence protein metabolism volunteered to participate in the study. All procedures complied with the ethical standards outlined in the Tri‐Council Policy statement for research involving humans and were approved by the Hamilton Integrated Research Ethics Board (REB Project #14‐333 and #2867 for the methodological and validation trials, respectively). Prior to participation, all subjects read and signed an informed consent form.

### 2.3 Experimental protocol

**Figure 1:**
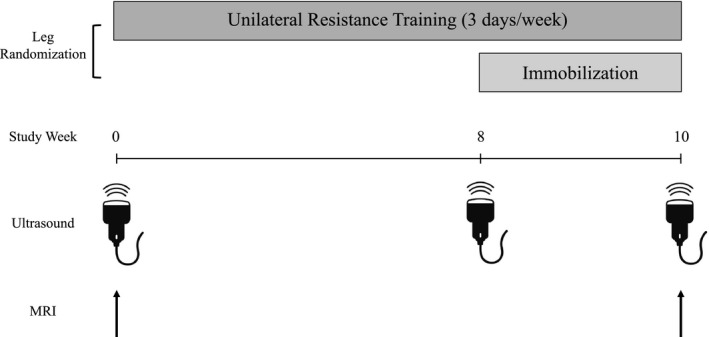
Schematic overview of the study. Subjects had one leg randomly assigned to perform 10 weeks of unilateral resistance exercise training three days per week. The contralateral leg performed habitual activities during the first 8 weeks of the study and was immobilized from week 8–10

For a schematic overview of the main experimental design, refer to Figure 1. Participants performed 10 weeks of unilateral resistance training, which consisted of thrice weekly leg press and leg extension training sessions under supervision. During the last two weeks of the study, a knee brace (DonJoy) was applied to the contralateral limb to induce immobilization as previously described (Holloway et al., 2019). Mid‐thigh VLCSA was assessed before (day 0) and after the study (day 70) using B‐mode ultrasonography (VLCSA_US_) and MRI (VLCSA_MRI_). An additional ultrasound scan of VLCSA was obtained at week 8 to confirm that no changes in muscle size had occurred in the free‐living limb prior to undergoing immobilization. Muscle volume and peak quadriceps CSA were also measured using MRI at day 0 and day 70.

### 2.4 Unilateral resistance exercise

Following a brief warmup on a cycle ergometer, participants performed three sets of unilateral leg extension followed by three sets of leg press. Loading was set at ~80% of 1‐RM, which was predetermined using three successive familiarization sessions prior to commencing the study. This intensity led to muscle failure between 8 and 12 repetitions (defined as an inability to execute another repetition through a full range of motion). If the participant successfully completed less than 8 or more than 12 repetitions, the load for subsequent sessions was adjusted accordingly. After each exercise session, participants ingested 25 g of whey protein isolate (Leprino Foods, Denver, CO, USA).

### 2.5 Immobilization

Immobilization was accomplished by applying an X‐ACT ROM knee brace (DonJoy, Vista, CA, USA) on the contralateral non‐training leg during the last two weeks of the study (i.e., weeks 9 and 10). Prior to brace application, the leg randomly assigned to be immobilized was only subjected to activities of daily living. The angle of the brace was adjusted to permit toe clearance during ambulation with crutches without *active* hamstring flexion (~60° of flexion). The angle was subsequently locked into place with tabs provided by the manufacturer and secured with ties to prevent angle unlocking. Tape was wrapped around the brace and an investigator's signature was written such that if the brace was removed, the tape would be damaged. The brace was briefly removed at each exercise visit by an investigator and the (unweighted) leg was inspected for signs of a deep vein thrombosis following a procedure previously described (Oates et al., 2010). There were no reports of swelling or pain in the immobilized limb, and only minor skin chaffing occurred.

### 2.6 Magnetic resonance imaging

Participants underwent a fasted MRI scan of both thighs on day 0 and upon study completion (day 70) for the assessment of mid‐thigh VLCSA, peak quadriceps CSA and muscle volume. Each scan was performed in a 3‐Tesla HD Scanner (SIGNA MRI System; GE Medical, Milwaukee, Wisconsin) at the Imaging Research Center (St. Joseph's Healthcare, Hamilton, ON). Participants rested in a supine position for ~10–15 minutes prior to scanning to normalize fluid shifts in the body. Axial (transverse) MR images were obtained from both thighs from the distal end of the femur to greater trochanter. A fast‐recovery, fast spin‐echo pulse sequence was used, along with iterative decomposition of water and fat with echo asymmetry and least‐squares estimation post‐processing to obtain water‐only, fat‐only, in‐phase, and out‐of‐phase images of the thighs. The following parameters were used: repetition time = 2000 msec, echo time=30 msec, refocusing flip angle = 111 degrees, echo train length = 6, Array coil spatial sensitivity encoding (parallel imaging factor) = 2, field of view = 42x21 cm, acquisition matrix = 512 × 256, 3 mm slice thickness, and 0 mm slice gap. An average of 140 slices was acquired from each participant. The acquisition was completed in two sections: a lower stage and an upper stage, which were subsequently stitched together by an MR technician. The total scan time for both stages was approximately 11 min.

Images were downloaded from a secure server and VLCSA was manually traced using the polygon selection tool in ImageJ. For comparisons with the ultrasound, mid‐thigh VLCSA was analyzed from the image slice corresponding to 50% of the distance between the greater trochanter and lateral epicondyle of the femur, identified using a coronal image as a reference. Peak quadriceps CSA (i.e., the cross‐sectional MRI image slice for which the quadriceps CSA was maximal) and quadriceps volume were quantified using semi‐automatic software, Analyze 14.0 (AnalyzeDirect, Overland Park, NS) as previously described (Holloway et al., 2019). First, the distal 20% of thigh images and the proximal 30% of MRI images from each leg were excluded from analysis due to an increased difficulty to differentiate muscle groups at these extremes. Total quadriceps CSA was subsequently traced manually on every other image slice. The tracings were propagated to untraced images and used to train an algorithm to differentiate muscle from adipose and connective tissue. These images were in turn randomly checked for accuracy. Muscle volume (cm^3^) was calculated as the sum of CSA values from all of the analyzed image slices multiplied by the slice thickness.

### 2.7 Ultrasonography

Upon arrival to the laboratory after a ~ 10 h overnight fast, participants lied supine for 10 minutes to normalize fluid shifts in the body, while their feet were positioned in a custom foot‐hold apparatus that prevented depression of the thigh against the bed. During the first assessment, a mark was made with permanent ink at a point equidistant between the greater trochanter of the femur and the lateral epicondyle of the knee that was identified by palpation. The medial border of the *vastus lateralis* was identified along this point and a second mark was made on the leg. A straight line was then drawn down the leg perpendicular to the surface of the bed with horizontal markings made every 2 cm, which served as a guideline for ultrasound probe placement. Transparent paper was used to trace this guideline and any identifiable marks on the skin (moles, birthmarks, scars, etc.) that could be used as reference points for subsequent scans. To acquire images, a 50 mm 12.5 linear‐array ultrasound probe (Vivid Q, GE Medical Systems, Horten, Norway) was positioned at the start of the *vastus lateralis*.

For the methodological trial, muscle thickness measurements were obtained with the probe oriented along the longitudinal axis of the *vastus lateralis*. To standardize the amount of force applied on the leg, the ultrasound probe was fixed—via a 90° angle bracket—to a strain gauge that displayed force (Dillon Model GL, Fairmont, MN, USA). This apparatus was in turn fixed to a vertical test stand with a movable clamp that could be modified based on the size of the participant. Four muscle thickness measurements were subsequently obtained from each participant (one at 0.2, 0.5, 0.7, and 1.0 Newtons (N) of applied force).

For the validation trial, a series of ~4–6 overlapping images were acquired from the start to the end of the *vastus lateralis*. To prevent depression of the muscle belly by the probe—in accordance with data generated in our pilot work—and to ensure adequate acoustic contact with the skin, a thick layer of water‐based gel (~2 cm) was applied at the region of scanning and the probe was rested on top. Muscle thickness and muscle CSA images were converted from DICOM to.jpeg format using DICOM editing software (Sante DICOM Editor, version 3.1.2, Athens, Greece) and randomized by assigning to each image a letter and number identifier (i.e., “A56”). Muscle thickness was quantified as the linear distance from the superficial and deep fascial borders of the *vastus lateralis*. Images for CSA analysis were stitched together using open‐source image editing software (GNU Image Manipulation Program v. 2.8.22, Mountain View, CA, USA) by aligning subcutaneous fat, superficial and deep aponeuroses, and intramuscular fat deposits between subsequent images and then traced using the polygon tool in ImageJ.

### 2.8 Statistical analyses

All statistical analyses were performed using SPSS Statistics for Mac, version 23.0 (IBM Corp., Armonk, NY, USA) with the exception of sample size, which was calculated using “pwr.r.test” from the “pwr” package in R (version 4.02). Specifically, to achieve an effect size of 0.69 (Pearson's r obtained from Franchi, Longo, et al., 2018) at a significance level of 0.05 and power of 80%, 13 participants were required. Changes in muscle thickness over time and at different probe pressures were measured using a two‐way repeated measures ANOVA. VLCSA_US_ was analyzed using a two‐way repeated‐measures analysis of variance (ANOVA) with time (3) and leg (2) as within‐subjects’ factors. Changes in VLCSA_MRI_ were analyzed using a two‐way repeated measures ANOVA with time (2) and leg (2) as within‐subjects’ factor. If the ANOVA test revealed a significant interaction, a Tukey's HSD post hoc test was employed to interrogate pairwise differences between legs and across time points. Pearson's correlations were run to assess the relationship between the various measurements of muscle size (% change from pre‐ to post‐ intervention) obtained by ultrasonography and MRI. Bland‐Altman plots were constructed to assess the agreement between ultrasonography with MRI. Inter‐ and intra‐rater reliability of VLCSA_US_ image analyses were examined using a random series of 36 images and a two‐way random‐effects model with absolute agreement [i.e., ICC(2,2)] (Koo & Li, 2016). Two investigators (T.S and T.T) independently stitched together and traced pre‐ and post‐intervention images to assess inter‐rater reliability. One investigator analyzed a randomized series of the same images from the same subjects ~8 weeks apart for the measurement of intra‐rater reliability. Interpretation of the strength of each ICC was determined by referencing recommended threshold values (Koo & Li, 2016). We also calculated the 95% Minimal Detectable Change (MDC) using the equation discussed in (Weir, 2005):

$$\text{MDC}\left(95\%\right)=\text{SEM}*\sqrt{2}*1.96$$

where *SEM* (Standard Error of the Measurement) and is equal to the standard deviation of the change score between the first and second stitching trial.

Data are plotted as box plots showing the group mean and median, interquartile range (box), and maximum and minimum (whiskers) values. Data are reported as means ±standard deviation (SD), and results were considered statistically significant when *p* ≤ 0.05.

## 3 RESULTS

### 3.1 Methodological study

**Figure 2:**
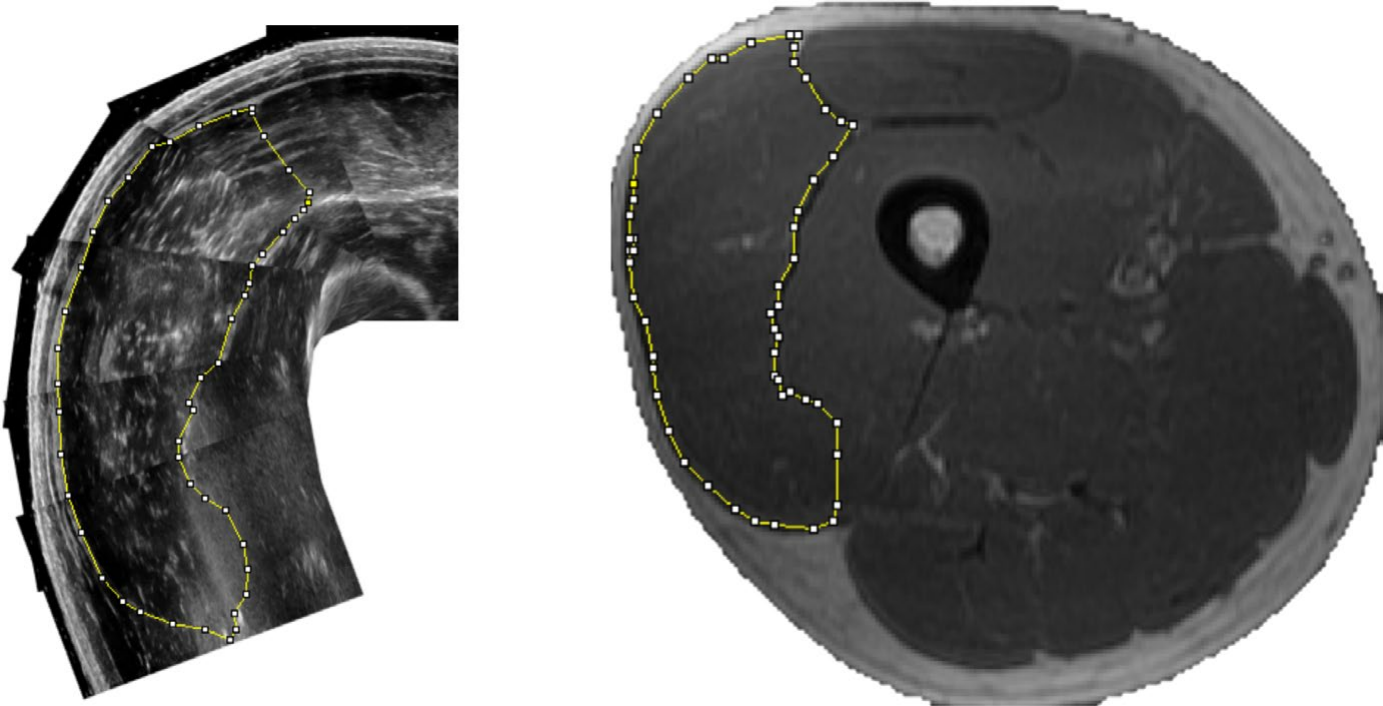
Representatives images of *vastus lateralis* CSA (VLCSA) obtained from (a) B‐mode ultrasonography, and (b) Magnetic resonance. VLCSA was manually outlined in both images using open‐source (ImageJ) software by the investigators in a randomized and blinded manner

We could not *consistently* obtain forces of 0 N. That is, even resting the probe on a relatively thick (~2 cm) layer of gel produced variations in applied forces ranging from 0 N to 0.2 N. Thus, to standardize force measurements, we set 0.2 N as the baseline “reference” force which, importantly, did not visibly depress the skin overlying the *vastus lateralis* at the point of measurement but ensured sufficient acoustic contact with the gel for image quality. At this applied reference force, muscle thickness pre‐RT was 28.8 ± 4.3 mm. However, muscle thickness values were significantly lower (*p* < 0.05) when 0.5 N (28.1 ± 4.4 mm), 0.7 N (27.6 ± 4.5), and 1.0 N (26.8 ± 4.4 mm) of force was applied. Moreover, significant differences in muscle thickness (*p* < 0.05) were observed when 1.0 N versus 0.2 N of force were applied at pre‐ and post‐ time points, respectively. We determined that this increase was likely due to the greater relative force (and thus the depression of the muscle) at the pre‐RT time point because, in the subset of subjects studied, we did not observe a significant increase in muscle thickness when both pre‐ and post‐RT measurements were obtained at a force of 0.2 N (*p* > 0.05). Thus, for the primary analysis (below) we elected to obtain CSA images by resting the ultrasound probe on the gel layer without depressing the underlying skin and muscle—which corresponded to an applied probe pressure of less than or equal to 0.2 N. Representative MRI and ultrasound images of the *vastus lateralis* are shown in Figure 2.

### 3.2 Validation study

#### 3.2.1 Participant Characteristics

Participant characteristics are displayed in Table 1. Briefly, 14 participants completed the intervention (21 ± 3 years old). We ascertained that two participants from the 14 were non‐compliant with the immobilization protocol (changed flexion angle of the brace, which enabled partial weight‐bearing, without authorization by study investigators), but were included in the analysis because we were only concerned with comparing muscle size measurements between US and MRI, not the degree of atrophy *per se*. Inclusion or removal of the non‐compliant participants from the analysis did not change the conclusions drawn from the data.

**Table 1:** Participant characteristics, n = 14

Parameter	Baseline	Week 5	Week 10
Age, y	21 ± 3	—	—
Height, m	1.72 ± 0.06	—	—
Mass, kg	72.4 ± 13.6	71.8 ± 14.0	72.2 ± 13.3
Daily Steps	10200 ± 4600	9000 ± 3000	8300 ± 3400
Activity, kcal·d^−1^	1113 ± 667	986 ± 569	1049 ± 564
Energy intake, kcal·d^−1^	2300 ± 550	2200 ± 850	2450 ± 820
Dietary Protein, g·kg·d^−1^	1.5 ± 0.9	1.5 ± 0.6	1.6 ± 0.9
En% Protein	17 ± 6	18 ± 5	18 ± 4
En% CHO	49 ± 12	51 ± 8	52 ± 10
En% Fat	33 ± 11	32 ± 8	30 ± 10

#### 3.2.2 Ultrasound Reliability and MDC

Intra‐rater reliability was good to excellent with an ICC of 0.940 (95% CI: 0.875 to 0.972). Inter‐rater reliability was moderate to excellent (ICC=0.847; 95% CI: 0.666 – 0.926). The SEM and MDC for our image stitching technique were 1.09 cm^2^ and 3.03 cm^2^, respectively.

#### 3.2.3 Mid‐thigh VLCSA_US_

**Figure 3:**
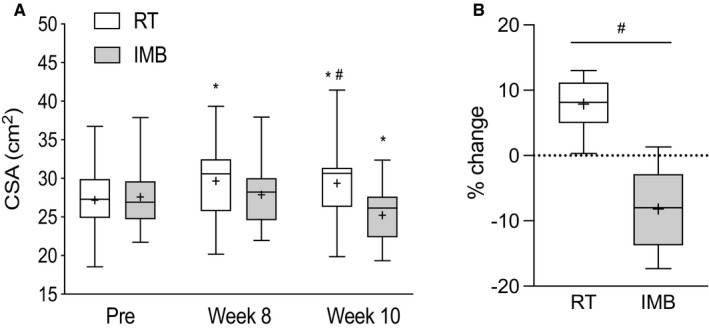
Box and whisker plots showing (a) absolute values of VLCSA and (b) percentage change in VLCSA after 10 weeks of RT and 2 weeks of IMB. Upper and lower boundaries of the boxes represent the 75th and 25th quartiles, respectively. The whiskers represent the maxima and minima. The central horizontal line represents the median and the cross represents the mean value. *denotes a statistical difference from Pre value in the respective leg and # denotes a significant difference between legs at a given time point (*p* < 0.05)

VLCSA_US_ data are presented in Figure 3. VLCSA_US_ did not differ between the RT and IMB limbs at baseline (*p* > 0.05). VLCSA_US_ increased from 27.2 ± 4.4 to 29.4 ± 5.2 cm^2^ after 10 weeks of RT (7.9 ± 3.8%; *p* < 0.05). There was no difference in VLCSA_US_ in the IMB leg from weeks 1 to 8, but VLCSA_US_ was significantly reduced from 27.6 ± 4.5 to 25.2 ± 3.6 cm^2^ in response IMB (weeks 8 to 10) (−8.2%±5.8%; *p* < 0.05).

#### 3.2.4 Mid‐thigh VLCSA_MRI_, peak VLCSA_MRI_, and muscle volume

**Figure 4:**
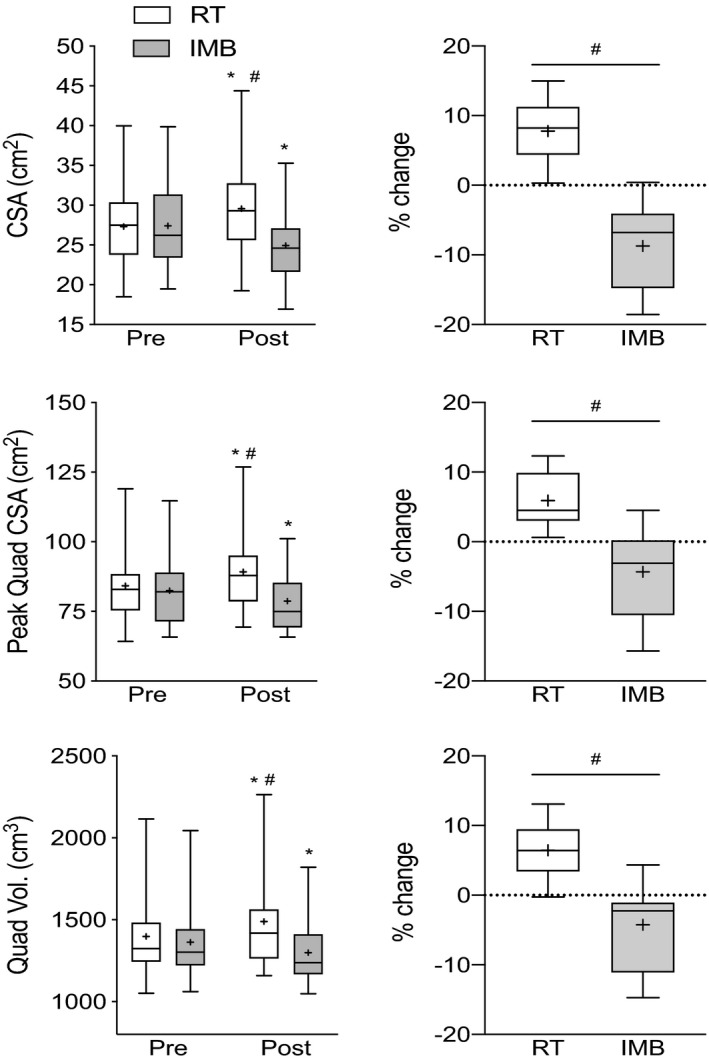
Box and whisker plots showing (a) VLCSA, (b) peak quadriceps CSA, and (c) quadriceps muscle volume before and after RT (open box and whisker plots) and IMB (gray box and whisker plots). Upper and lower boundaries of the boxes represent the 75th and 25th quartiles, respectively. The whiskers represent the maxima and minima. The central horizontal line represents the median and the cross represents the mean value. *denotes a statistical difference from Pre value in the respective leg and # denotes a significant difference between legs at a given time point (*p* < 0.05)

VLCSA_MRI_ data are presented in Figure 4a. VLCSA_MRI_ increased from 27.3 ± 5.3 to 29.6 ± 6.4 cm^2^ after RT (7.8 ± 4.5%; *p* < 0.05) and decreased from 27.4 ± 5.3 to 24.9 ± 4.5 cm^2^ after IMB (−8.7 ± 6.1%; *p* < 0.05). Peak quadriceps CSA (Figure 4b) and quadriceps volume (Figure 4c) increased significantly by 5.9 ± 3.9% and 6.4 ± 3.7% after RT, respectively. Relative to Pre‐IM, peak quadriceps CSA (−4.3 ± 6.3%) and muscle volume (−4.3 ± 6.0%) were both significantly reduced in response to IMB (*p* < 0.05).

#### 3.2.5 Correlations

**Figure 5:**
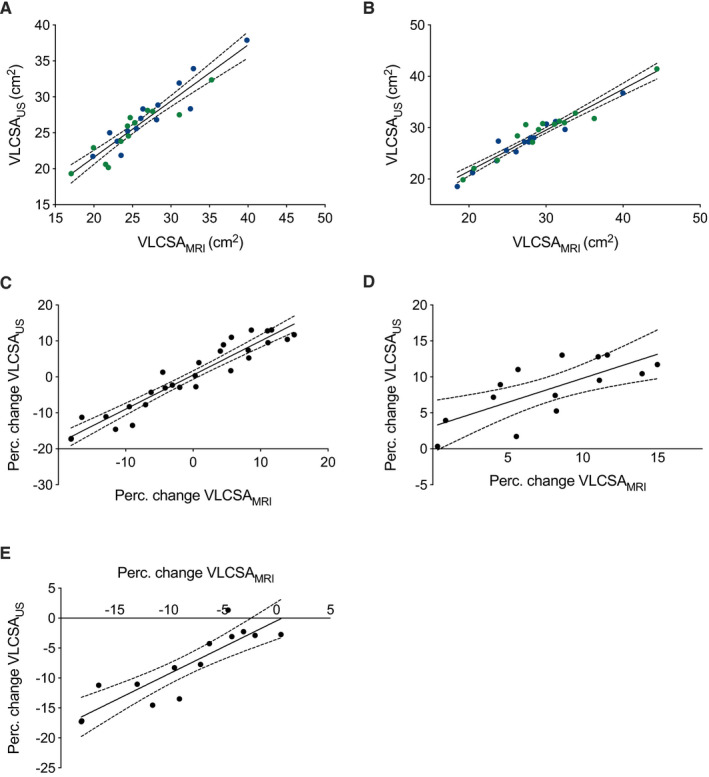
(a and b) Scatterplots showing the relationship between absolute VLCSA_US_ and VLCSA_MRI_ measurements taken from the IMB leg (a) and RT leg (b). Blue dots depict baseline values, whereas green dots represent values taken at week 10. (c‐e) Scatterplots showing the relationship between the percentage change in (c) VLCSA_US_ and VLCSA_MRI_ including both RT and IMB limbs, (d) VLCSA_US_ and VLCSA_MRI_ in the limb subjected to resistance training (RT), and (e) VLCSA_US_ and VLCSA_MRI_ in the limb subjected to immobilization (IMB). The solid line and the dotted lines in Panels A through E represent the line of best fit and the 95% confidence intervals, respectively

Statistically significant correlations were observed between VLCSA_US_ and VLCSA_MRI_ at baseline and 10 weeks in accordance with previous research(Franchi, Longo, et al., 2018; Lixandrao et al., 2014) (r > 0.85 for all comparisons; Figures 5a and b). Furthermore, there were strong correlations between the percentage change in VLCSA_US_ and VLCSA_MRI_ (r = 0.95; Figure 5c), peak quadriceps CSA (r = 0.82; data not shown), and quadriceps muscle volume (r = 0.83; data not shown) when the data from both legs were combined. When data were analyzed separately by intervention, the correlations between the percentage change in VLCSA_US_ and VLCSA_MRI_ (r = 0.85; Figure 5d), peak quadriceps CSA (r = 0.65; data not shown), and muscle volume (r = 0.67; data not shown) in the IMB limb remained significant. After RT, however, only the percentage change in VLCSA_US_ remained significantly correlated with VLCSA_MRI_ (r = 0.67; Figure 5e).

#### 3.2.6 Bland‐Altman Plots

**Figure 6:**
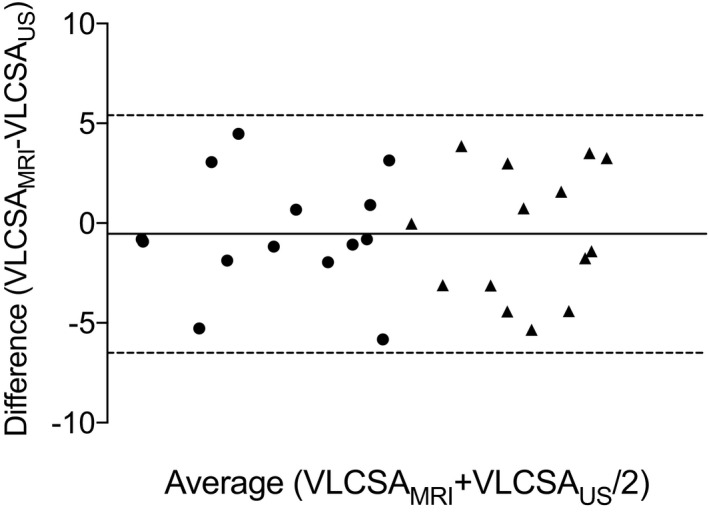
Bland‐Altman plot showing the methodological agreement between the percentage change in VLCSA_US_ and VLCSA_MRI_ after 10 weeks of resistance training (triangles) and 2 weeks of immobilization (circles). Dotted horizontal lines represent the upper and lower limits of agreement and the solid horizontal line represents the bias of ultrasonography relative to MRI

A Bland‐Altman plot showing the agreement between percentage change in VLCSA_US_ and VLCSA_MRI_ in response to RT and IMB is displayed in Figure 6. There was minimal bias between methods for the measurement of the percentage change in VLCSA (−0.5 ± 3.0%; *p* > 0.05). All measured values fell within the upper (5.4%) and lower (−6.5%) limits of agreement for both interventions with no evidence of bias within the range of measured values.

## 4 DISCUSSION

We report for the first time that ultrasound‐derived measurements of VLCSA hypertrophy and atrophy induced by resistance training and immobilization are significantly correlated with MRI‐derived measurements when the appropriate care is taken to avoid depression of skin and muscle. Moreover, we showed that although changes in VLCSA_US_ did not correlate with changes in muscle volume in response to RT, there was a significant relationship between these indices in response to immobilization. The reliability of our ultrasound assessment technique was in the range of good to excellent, and the bias compared to MRI was minimal. These results suggest that B‐mode ultrasonography is a suitable alternative for the assessment of muscle hypertrophy and muscle atrophy under conditions of dynamic loading in young men.

MRI is considered to be the gold‐standard for non‐invasively measuring muscle size. However, it is cost‐prohibitive, which has limited its routine use in physiological research. We report that, with practice and skilled operation, ultrasonography offers comparable tissue differentiation capabilities but it cannot quantify large muscles like the *vastus lateralis* in a single image because of the field of view limitation of US probes (Esformes et al., 2002; Franchi, Raiteri, et al., 2018). The introduction of the extended‐field‐of‐view ultrasound technique (EFOV; also called panoramic ultrasound), which relies on algorithms to automatically overlap ultrasound images in real‐time, has been proposed as a solution and alternative to the time‐consuming process of stitching images together manually. This method has demonstrated a high degree of concordance with MRI when measuring hamstrings CSA in an athletic cohort (Franchi et al., 2020), quadriceps muscle hypertrophy in response to RT (Ahtiainen et al., 2010), and muscle atrophy in response to bed rest (Scott et al., 2017). However, the EFOV method is limited by the relative difficulty in applying constant (and minimal) probe pressure throughout the scanning process (Ahtiainen et al., 2010; Franchi, Raiteri, et al., 2018), unless specially designed equipment is used to control probe trajectory (Noorkoiv et al., 2010). In our view, probe pressure is easier to control using the manual stitching technique that we elected to use, which allows the operator to reset his/her posture after capturing each image.

The technique of stitching several images together to overcome the small field of view of traditional ultrasound probes was developed ~15 years ago and was shown to provide valid measurements of VLCSA in a small, homogenous population of elderly adults (Reeves et al., 2004). Since this pioneering work, other research groups have confirmed the applicability and cross‐sectional validity of this technique in larger sample cohorts with a wider range of *vastus lateralis* CSA (Franchi, Longo, et al., 2018; Lixandrao et al., 2014). Our data are in broad agreement with these data (Franchi, Longo, et al., 2018; Lixandrao et al., 2014), confirming the accuracy of a single assessment of muscle CSA using ultrasound. More recently, it was shown that obtaining one‐dimensional muscle thickness measurements using ultrasound, obviating the need for serial image stitching, also correlates strongly with MRI‐derived measurements of VLCSA and muscle volume (Franchi, Longo, et al., 2018). Together these findings provide support for the use of ultrasound to assess muscle size at a single‐time point, which has important clinical utility such as effectively differentiating sarcopenic from non‐sarcopenic older persons (Ismail et al., 2015). However, the ability to accurately measure *changes* in muscle size is arguably more important, as doing so allows researchers to test the efficacy of dietary or exercise interventions or clinical conditions that result in either hypertrophy or atrophy.

The use of US to quantify muscle hypertrophy in response to resistance training is a relatively common practice and has led to important insights regarding regional hypertrophy and other morphological adaptations (i.e., changes in the pennation angle and fascicle length Earp et al., 2015; Mangine et al., 2018; Wells et al., 2014). However, very few studies have evaluated the validity of ultrasound against the reference standard to accurately measure muscle growth. Franchi *et al* demonstrated a significant correlation between changes in muscle thickness assessed by ultrasound after 12 weeks of unilateral RT and the corresponding changes in VLCSA measured by MRI, indicating that US‐derived MT measurements could serve as a useful surrogate for mid‐thigh VLCSA hypertrophy (Franchi, Longo, et al., 2018). However, changes in MT were not correlated with changes in muscle volume (Franchi, Longo, et al., 2018). In accordance with these findings (Franchi, Longo, et al., 2018), we observed a significant correlation between ultrasound‐ and MRI‐measured changes in mid‐thigh VLCSA, but not between VLCSA and muscle volume in response to RT. The reasons for this lack of relationship are unclear but could be due to regional differences in muscle hypertrophy (Earp et al., 2015; Franchi et al., 2014; Franchi, Longo, et al., 2018; Mangine et al., 2018; Wells et al., 2014). Indeed, changes in fascicle length—which have been reported to occur—are more likely to affect the growth of distal regions of the muscle (Jorgenson et al., 2020) and would, therefore, be undetected by a single measurement at the mid‐point of the thigh.

To circumvent the potential confounding influence of regional hypertrophy, and strengthen the association between ultrasound and MRI in future investigations, multiple images could be obtained along the length of the muscle and volume estimated using the Cavalieri principle (Infantolino et al., 2007). Indeed, the measurement of *vastus lateralis* muscle volume using ultrasound is largely concordant with volume estimates derived from the hydrostatic weighing of cadaver samples when applying this method (Infantolino et al., 2007). Again, however, these measurements were made at a single‐time point (Infantolino et al., 2007) and the addition of repeated measurements to assess changes over time will inevitably introduce additional error and likely weaken the association. These errors are likely to be magnified by not taking into account the impact of probe pressure during serial image acquisition. Thus, future research is warranted to investigate whether the derivation of muscle volume from a series of US images would strengthen the relationship between US and MRI when assessing muscle hypertrophy.

In comparison to resistance exercise, there is a paucity of literature assessing the efficacy of ultrasound to detect, and accurately measure, changes in muscle size associated with disuse and muscle‐wasting conditions. Puthucheary and colleagues showed that the loss of *rectus femoris* CSA measured by ultrasound correlated with the extent of organ failure and length of stay in critically ill patients admitted to the ICU (Puthucheary et al., 2013). Thus, although the authors did not formally assess the relationship between ultrasound and MRI measured muscle loss in these patients, the correlations identified between ultrasound‐derived measurements of muscle size and clinically relevant outcomes provided evidence that ultrasound might be useful for quantifying skeletal muscle loss in a patient population (Paris et al., 2017; Puthucheary et al., 2013). Scott *et al* recently validated the use of panoramic ultrasound against MRI for measuring changes in VLCSA in response to bed‐rest with or without an exercise counter‐intervention reporting close agreement between methods (Scott et al., 2017). In line with their findings, we also observed a close association between methods using image‐stitching using an ultrasound unit lacking automated panoramic capabilities. We also provide novel data showing that the loss of VLCSA in response to immobilization measured by US is correlated with the reduction in muscle volume measured by MRI. These data suggest that, in contrast to muscle hypertrophy, muscle atrophy appears to be more homogenous through the length of the muscle in response to immobilization. However, whether ultrasound can detect early stage smaller decreases in muscle size in response to shorter term disuse (i.e., 3–7 days) requires further investigation.

### 4.1 Limitations

The primary limitation of the present study is that we only measured the *vastus lateralis* muscle at a single site using US. There is evidence that the quadriceps muscle group exhibits regional muscle hypertrophy (Franchi et al., 2014; Franchi, Longo, et al., 2018; Mangine et al., 2018; Wells et al., 2014), which would be missed when only assessing the mid‐thigh region of one muscle. Indeed, as we discussed above, this may be a major reason for the lack of correlation we observed between VLCSA measurements by US and MRI‐derived assessments of muscle volume in response to RT. Obtaining multiple measurements of VLCSA at different regions of the thigh and deriving an estimate of muscle volume would, we hypothesize, strengthen the association between methods. Second, while the average changes in VLCSA observed in our study exceed the minimal detectable change of 3.03 cm^2^ we calculated, our stitching technique may not be suitable to detect more subtle changes in muscle size that occur with other interventions that induce comparatively minor changes in VLCSA. Finally, our analysis included only younger, college‐aged men, a population with a relatively thin layer of subcutaneous adipose tissue overlying the muscle. Future research is needed to determine how US compares to MRI in older populations and in overweight/obese individuals with thicker subcutaneous adipose depots that may reduce the accuracy of our image stitching technique.

## 5 CONCLUSIONS

We show that B‐mode ultrasonography provides a comparable alternative to magnetic resonance imaging for measuring changes in muscle size in response to loading‐induced hypertrophy and immobilization‐induced atrophy of the *vastus lateralis* in young men. In addition, we highlight that it is important to not depress the skin during image acquisition, and that caution is warranted when comparing ultrasound‐derived muscle CSA and MRI‐derived muscle volume. Further work is required to establish that this conclusion is valid in women and in older persons or other clinical scenarios.

## CONFLICTS OF INTEREST

The authors declare no conflicts of interest.

## FUNDING INFORMATION

TS was funded by the Ontario Graduate Scholarship during the conduct of this study. This work was supported by a National Science and Engineering Research Council (NSERC) of Canada Discovery grants to SMP and MJM and a National Science and Engineering Research Council (NSERC) of Canada Research Tools and Instruments grants to MJM.
